# Prognostic factors of patients with left-sided obstructive colorectal cancer: post hoc analysis of a retrospective multicenter study by the Japan Colonic Stent Safe Procedure Research Group

**DOI:** 10.1186/s12957-022-02490-9

**Published:** 2022-01-27

**Authors:** Shungo Endo, Noriyuki Isohata, Koichiro Kojima, Yoshihiro Kadono, Kunihiko Amano, Hideo Otsuka, Tatsuya Fujimoto, Hideto Egashira, Yoshihisa Saida, the Japan Colonic Stent Safe Procedure Research Group, K. Takayasu, M. Ushigome, M. Ebi, Y. Sumida, S. Asai, K. Nasu, T. Shiratori, T. Kawamura, T. Ohki, H. Naota, H. Matsushita, N. Watanabe, A. Kohyama, T. Kuwai, S. Saito, K. Ohta, T. Kimura, O. Okamura

**Affiliations:** 1 Department of Coloproctology, Aizu Medical Center, Fukushima Medical University, Aizu-wakamatsu, Japan; 2 Department of Surgery, Kyorin University, Tokyo, Japan; 3 Department of Gastroenterology, Saiseikai Kumamoto Hospital, Kumamoto, Japan; 4 Department of Digestive Tract and General Surgery, Saitama Medical Center, Saitama Medical University, Saitama, Japan; 5 Department of Surgery, Tokyo Metropolitan Tama Medical Center, Tokyo, Japan; 6 Department of Gastroenterology, Kimitsu Chuo Hospital, Chiba, Japan; 7 Department of Gastroenterology, Shonan Kamakura General Hospital, Kanagawa, Japan; 8 Department of Surgery, Toho University Ohashi Medical Center, Tokyo, Japan

**Keywords:** Obstructive colorectal cancer, Emergency surgery, Self-expandable metallic stent, Relapse-free survival, Prognostic factor, Stage migration

## Abstract

**Background:**

There are many reports on the choice of treatment for and prognosis of left-sided obstructive colorectal cancer; however, few studies have focused on the prognostic factors of left-sided obstructive colorectal cancer. Therefore, we analyzed the prognostic factors using a post hoc analysis of a retrospective multicenter study in Japan.

**Methods:**

A total of 301 patients were enrolled in this study to investigate the prognostic factors for relapse-free survival. The relationships between sex, age, decompression for bridge to surgery, depth of invasion, lymph node metastasis, postoperative complications, adjuvant chemotherapy, carcinoembryonic antigen, carbohydrate antigen 19-9, neutrophil-to-lymphocyte ratio, and relapse-free survival were examined.

**Results:**

No change in the decompression method, T3 cancer, negative postoperative complications (grades 0–1 of Clavien-Dindo classification), and adjuvant chemotherapy during Stage III indicated a significantly better prognosis in a Cox univariate analysis. Lymph node metastasis was not selected as a prognostic factor. Excluding patients with <12 harvested lymph nodes (possible stage migration), lymph node metastasis was determined as a prognostic factor. In a Cox multivariate analysis, change in the decompression method, depth of invasion, lymph node metastasis (excluding N0 cases with <12 harvested lymph nodes), and adjuvant chemotherapy were prognostic factors.

**Conclusions:**

Similar to those in nonobstructive colorectal cancer, depth of invasion and lymph node metastasis were prognostic factors in left-sided obstructive colorectal cancer, and patients with <12 dissected lymph nodes experienced stage migration. Stage migration may result in disadvantages, such as not being able to receive adjuvant chemotherapy.

## Introduction

Colorectal cancer (CRC) is the third most commonly diagnosed malignancy worldwide, accounting for approximately 1.4 million new cases per year. CRC is the third most common cancer in men (746,000 cases, 10.0% of the total) followed by women (614,000 cases, 9.2% of the total) and is the fourth leading cause of cancer-related deaths worldwide, with nearly 700,000 deaths in 2012 [1, 2].

Large bowel obstruction (15–30% of CRCs) represents approximately 80% of emergencies related to CRC, while perforation (1–10% of CRC cases) accounts for the remaining 20% [3–6]. The most common location of obstructive CRC is the sigmoid colon, with 75% of the tumors located distal to the splenic flexure [7]. Obstructive right-sided colon cancer is usually treated through emergency surgery with primary resection and ileocolic anastomosis [8]; however, it is controversial whether emergency or radical surgery after decompression for “bridge to surgery (BTS)” for left-sided obstructive colorectal cancer (LOCRC) should be considered [9]. The short-term outcomes of BTS using self-expandable metallic stents (SEMSs) are excellent, but long-term oncological outcomes are questionable. However, in recent years, an increasing number of reports have shown that the long-term outcomes of emergency and elective surgeries after decompression using SEMS are comparable [10–13].

Although there are many reports on the choice of treatment and prognosis of LOCRC, there are only a few studies on the prognostic factors of LOCRC [14]. Therefore, we analyzed the prognostic factors of LOCRC using post hoc analysis of a retrospective multicenter observational study in Japan that compared the survival and perioperative outcomes of colonic stenting and transanal decompression tube (TADT) placement with emergency surgery for LOCRC (CODOMO study) [15].

## Methods

### Study design and participants

**Fig. 1:**
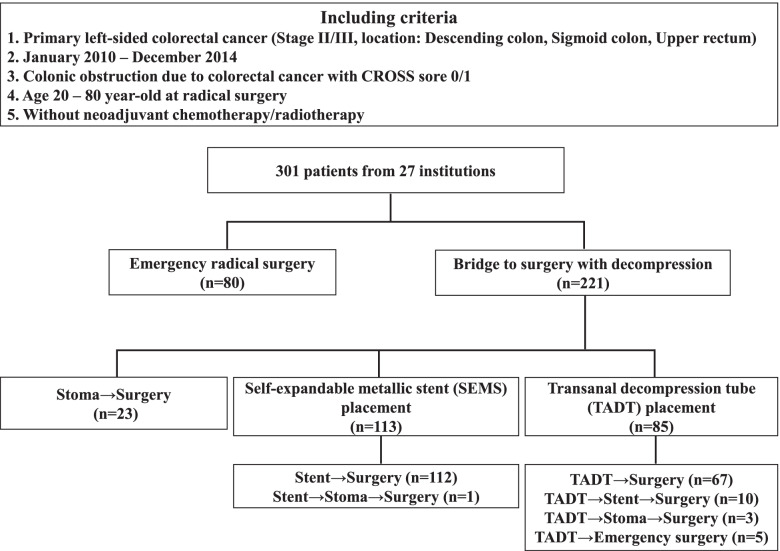
Study participants selection flowchart of CODOMO study

The CODOMO study was conducted by the Japan Colonic Stent Safe Procedure Research Group; the study design, eligibility criteria, and treatment parameters have been reported previously [15]. This study was conducted as a post hoc analysis of the CODOMO study to investigate prognostic factors for relapse-free survival (RFS) in LOCRC. The medical ethics committee of Fukushima Medical University reviewed and approved the observational study design, and the requirement for informed consent was waived. This study was also registered in the Japan University Hospital Medical Information Network Clinical Trial Registry (UMIN000024488). A summary of the previous study is as follows: the CODOMO study was a retrospective, multicenter, observational study comparing RFS and perioperative outcomes of colonic stenting, TADT placement, and emergency surgery for LOCRC. The participants were patients with histologically proven Stage II/III left-sided colon or upper rectal cancer with obstruction, who underwent subsequent surgery with curative resection between January 2010 and December 2014. The definition of the obstruction was specified based on the ColoRectal Obstruction Scoring System (CROSS) [14], and patients with CROSS 0 (requiring continuous decompression) and CROSS 1 (no oral intake) were included. The patients’ ages ranged from 20 to 80 years, and those treated with neoadjuvant chemotherapy and/or radiation therapy were excluded. Subsequently, 301 patients from 27 institutions met the inclusion criteria. Patients were divided into three groups based on decompression procedures: the surgery group with decompression by colostomy or intraoperative decompression during radical surgery (emergency surgery, *n* = 103), SEMS group using SEMS for BTS (*n* = 113), and TADT group with decompression using TADT for BTS (*n* = 85). There were 23 patients in the surgery group who underwent two-step surgery for stoma creation and curative resection for LOCRC and 19 patients with changes in decompression method (Fig. 1). In conclusion, the CODOMO study showed that patients who underwent SEMS placement for LOCRC had similar oncological outcomes to those who underwent emergency surgery; the TADT placement for BTS showed significantly lower RFS than those who underwent emergency surgery. Additionally, the total number of complications after curative surgery was significantly lower in the SEMS group than in the surgery group.

### Prognostic factors

In this study, the prognostic factors of LOCRC that influence RFS were analyzed. Among the aggregated data, the relationships between sex, age, decompression for BTS, change in decompression method, depth of invasion, lymph node metastasis, postoperative complications, adjuvant chemotherapy, preoperative serum carcinoembryonic antigen (CEA) level, preoperative serum carbohydrate antigen (CA 19-9) level, preoperative neutrophil-to-lymphocyte ratio (NLR), and RFS were examined. Patients were divided according to CEA level ≤ 5 ng/mL or > 5 ng/mL, and CA 19-9 level ≤ 37 U/mL or > 37 U/mL to examine their associations with RFS. NLR was divided into the following cutoff levels: ≤ 3.0 and >3.0, ≤ 5.0, and > 5.0. We adopted two cutoff values because there is no fixed cutoff value for NLR as a prognostic factor: some studies have a cutoff value of 3.0 [16, 17], whereas others have a cutoff value of 5.0 [18, 19].

### Statistical analysis

Quantitative data are reported as median (range). All statistical analyses were performed using SPSS ver. 25 (IBM, Armonk, NY, USA). Chi-square tests (Fisher’s exact tests) were used to compare discrete variables. RFS analysis was conducted using the Kaplan–Meier method and log-rank test to determine the significance of the survival curves. To identify prognostic factors for RFS, the Cox proportional hazards model was used for univariate and multivariate analyses. Statistical significance was set at *p* < 0.05.

## Results

### Patient characteristics

The baseline characteristics of the patients are summarized in Table 1. In this study, all patients were Japanese, with a median age of 69 years and a range of 28 to 80 years. Most of the patients were generally well, with an Eastern Cooperative Oncology Group performance status of 0 to 1 in 87.0% of cases. Tumor locations were all in the left-sided colon, with 20.9% in the descending colon, 69.8% in the sigmoid colon, and 9.3% in the upper rectum. Decompression methods for BTS included stoma creation (7.6%), SEMS (37.5%), and TADT (28.2%); however, emergency surgery with intraoperative decompression was 26.6%. Complications during the decompression period were as follows: two perforated cases using SEMS, two perforated cases, and five migrated cases using TADT. Of the perforated cases using SEMS, one was perforated by a guidewire and operated on at day 42 with continued decompression, and the other was perforated 18 days after SEMS placement, but decompression was completed; therefore, elective surgery was performed. These two cases were classified as no change in the decompression method. Two cases of perforation using TADT were performed during the emergency operations. There were 19 patients with changes in the decompression method (one SEMS case and 18 TADT cases), including two perforated cases and two migrated cases with decompression using TADT (Fig. 1). There was only one case with T2, and the other cases were T3 or T4 in depth of invasion. To ensure high quality in staging colon cancer, international guidelines recommend histopathological evaluation of at least 12 lymph nodes [20]. The number of harvested lymph nodes should be ≥12 to prevent stage migration [21, 22], but <12 was dissected in 19.9% of cases. The administration rate of adjuvant chemotherapy for Stage III cases was significantly higher than that for Stage II cases. Postoperative complications were graded according to the Clavien–Dindo classification [23] of 0 to 1 in 72.8% of cases and 2 to 5 in 27.2% of cases. There were missing values for preoperative CEA in 10 patients, CA 19-9 in 12, and NLR in 56, owing to the retrospective design of this study. The positive rates of CEA (cutoff level: 5.0 ng/mL) and CA 19-9 (cutoff level: 37 U/mL) were 57.4% and 17.3%, respectively.

**Table 1:** Demographical characteristics of the study population

Variable	Category	*n*		(%)
Age (years), median (range)		69	(28–80)	
Gender	Male	176		(58.5)
	Female	125		(41.5)
Performance status (ECOG)	0	184		(61.1)
	1	78		(25.9)
	2	22		(7.3)
	3	14		(4.7)
	4	3		(1.0)
Tumor location	Descending colon	63		(20.9)
	Sigmoid colon	210		(69.8)
	Upper rectum	28		(9.3)
Decompression for BTS	Stoma	23		(7.6)
	SEMS	113		(37.5)
	TADT	85		(28.2)
	No	80		(26.6)
Change in decompression method	Yes	19		(6.3)
	No	282		(93.7)
Depth of invasion (TNM)	T2	1		(0.3)
	T3	187		(62.1)
	T4 (T4a / T4b)	113	(85 / 28)	(37.5)
Lymph node metastasis (TNM)	N0	164		(54.5)
	N1	109		(36.2)
	N2	28		(9.3)
Number of harvested lymph nodes	<12	60		(19.9)
	≥12	241		(80.1)
Stage (TNM)	II (IIA/IIB/IIC)	164	(100/48/16)	(54.5)
	III (IIIA/IIIB/IIIC)	137	(1/110/26)	(45.5)
Adjuvant chemotherapy*	Stage II	51		(31.1)
	Stage III	97		(70.8)
Postoperative complication (Clavien-Dindo classification)	0	217		(72.1)
	1	2		(0.7)
	2	47		(15.6)
	3	28		(9.3)
	4	3		(1.0)
	5	4		(1.3)
CEA (ng/mL)	Not measured	10		(3.3)
	≤5	124		(41.2)
	>5	167		(55.4)
CA 19-9 (U/mL)	Not measured	12		(4.0)
	≤37	239		(79.4)
	>37	50		(16.6)
NLR	Not measured	55		(18.3)
	≤3	113		(37.5)
	3<, ≤5	75		(24.9)
	>5	58		(19.3)

*ECOG* Eastern Cooperative Oncology Group, *BTS* bridge to surgery, *SEMS* self-expanding metallic stent, *TADT* transanal decompression tube, *CEA* carcinoembryonic antigen, *CA 19-9* carbohydrate antigen 19-9, *NLR* neutrophil-to-lymphocyte ratio, *Administration rate of adjuvant chemotherapy was significantly higher in Stage III than in Stage II by Fisher’s exact test

**Fig. 2:**
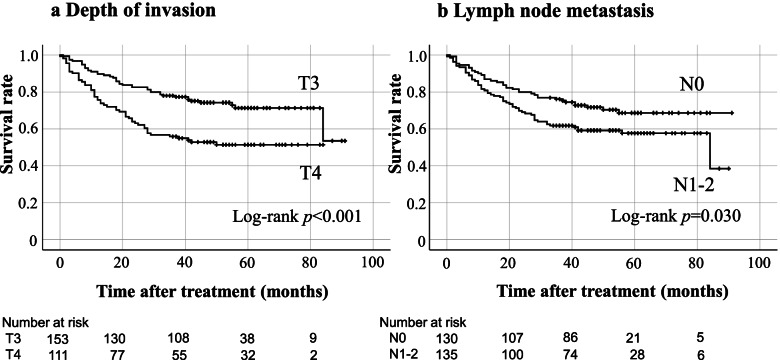
Kaplan-Meier curves presenting relapse-free survival rate in at risk patients with T3 or T4 depth of invasion (**a**) and that with N0 or N1-2 lymph node metastasis (**b**)

When we examined the relationship between the frequency of patients with <12 dissected lymph nodes and the status of preoperative decompression, patients who underwent emergency surgery without decompression presented with more cases with <12 dissected lymph nodes (Table 2). There were 34 cases with <12 harvested lymph nodes in lymph node metastasis-negative cases (N0) (13 cases of emergency radical surgery, 7 cases of stoma, 5 cases of SEMS, and 9 cases of TADT); therefore, cases with <12 harvested lymph nodes were excluded from the N0 cases in the analyses of prognostic factors because of the possibility of stage migration. Having ruled out the possibility of stage migration, lymph node metastasis was selected as a prognostic factor for LOCRC. The Kaplan–Meier curves for the depth of invasion and lymph node metastasis are shown in Fig. 2.

**Table 2:** Cases <12 harvested lymph nodes without/with preoperative decompression

	*n*	(%)	*p* value
Without preoperative decompression (*n*=80)	25	(31.3)	0.005
With preoperative decompression (*n*=221)	35	(15.8)	

*P* value was calculated by Fisher’s exact test

### Prognostic factors

The following prognostic factors were selected: performance status (Eastern Cooperative Oncology Group), tumor location, decompression for BTS, change in decompression method, depth of invasion, lymph node metastasis, postoperative complication, adjuvant chemotherapy, serum CEA level, serum CA 19-9 level, and NLR. Among these factors, no change in the decompression method, T3 cancer, negative lymph node metastasis (excluding lymph node metastasis-negative [N0] cases with <12 harvested lymph nodes), negative postoperative complications (grade 0–1 of the Clavien–Dindo classification), and administration of adjuvant chemotherapy during Stage III indicated a significantly better prognosis using Cox’s univariate analyses (Table 3).

**Table 3:** Prognostic factors by Cox’s univariate analysis for relapse-free survival

Variable	Survival (mo) Av. (95% CI)	HR	95% CI	*p* value
	Kaplan-Meier method	Cox’s univariate analysis		
Performance status (ECOG)				0.523
0–1 (*n*=262)	62.0 (57.4–66.5)	1		
2–4 (*n*=39)	59.9 (48.5–71.2)	0.845	0.505–1.416	
Location				0.381
Colon (*n*=273)	62.1 (57.6–66.5)	1		
Rectum (*n*=28)	57.7 (44.2–71.1)	0.381	0.433–1.377	
Decompression for BTS				0.155
No (*n*=80)	63.0 (56.0–70.0)	1		
Yes (*n*=221)	60.7 (55.6–65.7)	0.724	0.463–1.130	
Change in the decompression method				0.005
No (*n*=282)	63.8 (59.4–68.2)	1		
Yes (*n*=19)	35.5 (22.3–48.7)	0.422	0.232–0.769	
Depth of invasion				<0.001
T3 (*n*=179)	68.5 (63.3–73.7)	1		
T4 (*n*=121)	49.5 (43.2–55.9)	0.489	0.338–0.708	
Lymph node metastasis				0.177
N0 (*n*=164)	65.1 (59.5–70.7)	1		
N1-2 (*n*=137)	58.4 (52.1–64.8)	0.776	0.536–1.122	
Lymph node metastasis (excluding Stage II cases with <12 harvested lymph nodes)				0.036
N0 (*n*=130)	69.3 (63.4–75.1)	1		
N1-2 (*n*=137)	58.9 (52.5–65.2)	0.644	0.427–0.971	
Postoperative complication				0.026
grade 0-I (*n*=219)	64.9 (59.8–69.9)	1		
grade II-V (*n*=82)	54.0 (45.7–62.3)	1.553	1.053–2.289	
Adjuvant chemotherapy				0.616
No (*n*=153)	60.5 (54.6–66.4)	1		
Yes (*n*=148)	63.9 (58.0–69.8)	1.048	0.872–1.264	
Adjuvant chemotherapy in Stage II				0.214
No (*n*=113)	63.8 (58.0–69.6)	1		
Yes (*n*=51)	59.8 (49.1–70.6)	0.707	0.409–1.222	
Adjuvant chemotherapy in Stage III				0.003
No (*n*=40)	44.3 (32.6–56.0)	1		
Yes (*n*=97)	63.4 (56.7–70.0)	2.231	1.312–3.791	
Preoperative serum CEA level (ng/mL)				0.224
≤ 5 (*n*=124)	59.6 (54.2–65.0)	1		
> 5 (*n*=167)	59.4 (53.5–65.2)	0.787	0.535–1.158	
Preoperative serum CA 19-9 level (U/mL)				0.226
≤ 37 (*n*=239)	63.1 (58.3–68.0)	1		
> 37 (*n*=50)	53.4 (43.9–62.9)	0.748	0.468–1.196	
Preoperative NLR				
≤ 3 (*n*=113)	59.4 (53.0–65.9)	1		0.957
> 3 (133)	60.9 (54.3–67.5)	1.011	0.674–1.518	
≤ 5 (*n*=188)	59.2 (54.1–64.3)	1		0.798
> 5 (*n*=58)	61.8 (52.4–71.2)	1.064	0.660–1.716	

*Survival (mo) Av. (95% CI)* survival periods (months) average and 95% confidential interval, *HR* hazard ratio, *ECOG* Eastern Cooperative Oncology Group, *BTS* bridge to surgery, *SEMS* self-expanding metallic stent, *TADT* transanal decompression tube, *CEA* carcinoembryonic antigen, *CA 19-9* carbohydrate antigen 19-9, *NLR* neutrophil-to-lymphocyte ratio

There were 34 cases with <12 harvested lymph nodes in N0 cases; therefore, these cases were excluded from the N0 cases in the analyses of prognostic factors. Based on these results, we analyzed 266 cases, excluding one case of T2 and 34 cases of N0 with <12 harvested lymph nodes. Using Cox’s univariate and multivariate analyses, changes in the decompression method, T4 depth of invasion, lymph node metastasis-positive (excluding N0 cases with <12 harvested lymph nodes), and no adjuvant chemotherapy were poor prognostic factors (Table 4).

**Table 4:** Cox’s multivariate analysis for prognostic factors for relapse-free survival excluding Stage II cases with <12 harvested lymph nodes

Variable	Category	*n*	HR	95% CI	*p* value
Change in the decompression method	No/Yes	250/16	0.434	0.220–0.854	0.016
Depth of invasion	T3/T4	155/111	0.477	0.317–0.720	<0.001
Lymph node metastasis	N0/N1-2	130/136	0.514	0.324–0.815	0.005
Postoperative complication (CD classification)	0–1/2–5	71/195	0.681	0.442–1.050	0.082
Adjuvant chemotherapy	Yes/No	138/128	0.554	0.346–0.887	0.014

*HR* hazard ratio, *CI* confidential interval, *CD* Clavien-Dindo

## Discussion

Most studies on obstructive CRC report on the comparison with nonobstructive CRC and the efficacy of SEMS as BTS; there are few analyses on the prognostic factors of obstructive CRC. For BTS, there is no difference between decompression using a temporary stoma and SEMS [24]; many recent reports and meta-analyses show no difference in long-term outcomes between BTS using SEMS and emergency surgery [10, 12, 25]. Regarding BTS using TADT, small retrospective studies mostly from Japan showed no difference in outcomes from BTS using SEMS [26]. In this study, preoperative decompression had no effect on prognosis. However, new findings suggest that changes in the decompression method associated with perforation or poor decompression have a worse prognosis. Not all of the 10 cases of BTS using TADT that changed to stent failed to decompression; however, the decompression method was changed. Since the criteria for changes in the decompression method was ambiguous, we defined “change in the decompression method” in this study. As there was significant difference in the RFS between the group that had a change in the decompression method and the group that did not, we considered this category of “change in the decompression method” to be useful.

As prognostic factors for obstructive CRC, performance status, serum albumin level <4.0 g/dL, and resection of T4 and R1 cancers (cancer positive at cut end) were also independent risk factors for recurrence [14]. In a comparative study between emergency surgery and BTS using SEMS, age, performance status, depth of invasion, and lymph node metastasis were prognostic factors for disease-free survival [27]. A Japanese study of 50 cases of emergency surgery and 50 cases of BTS using SEMS for Stage II/III obstructive CRC showed that BTS and positive venous invasion were poor prognostic factors for RFS, and BTS and T4 cancer were poor prognostic factors for overall survival (OS) [28]. Long-term outcomes (OS and disease-free survival [DFS]) of emergency surgery and BTS using SEMS were equivalent, but NLR was a prognostic factor for DFS in emergency surgery. Additionally, the lymphocyte-monocyte ratio was a prognostic factor for OS and DFS of BTS using SEMS and OS in emergency surgery and BTS using SEMS [29]. Furthermore, a study of BTS cases using SEMS and TADT showed differences only in T4 cancer and the Controlling Nutritional Status score for DFS, with no differences in lymph node metastasis or adjuvant chemotherapy [30].

As mentioned above, for obstructive CRC, lymph node metastasis is not often selected. This may be because many reports on prognostic factors for obstructive CRC were based on a small number of cases and because of stage migration due to inadequate lymph node dissection or a small number of harvested lymph nodes during emergency surgery. Furthermore, in Stage II CRC, colonic obstruction is considered an independent poor prognostic factor [31], possibly weakening the impact of other prognostic factors.

In the analysis of all patients enrolled in this study, depth of invasion and postoperative complications were selected as prognostic factors; lymph node metastasis was not selected. When only Stage III cases were included, adjuvant chemotherapy was selected as a prognostic factor. Therefore, to exclude the possibility of stage migration due to insufficient lymph node dissection, we excluded patients with < 12 harvested lymph nodes in Stage II. Lymph node metastasis is a prognostic factor for patients with sufficient harvested lymph nodes. In Cox’s multivariate analysis, changes in the decompression method, depth of invasion, lymph node metastasis, and adjuvant chemotherapy were also selected as independent prognostic factors. The number of cases with <12 harvested lymph nodes was significantly higher in patients undergoing emergency surgery, possibly because of insufficient lymph node dissection due to general condition or bowel dilatation, or insufficient lymph node collection from the resected specimen after surgery. In Japan, collecting lymph nodes from the resected specimen is the surgeon’s responsibility; this may have been inadequate during emergency surgeries that are often performed after hours. Deference from Western guidelines, adjuvant chemotherapy for Stage II is not strongly recommended in the Japanese guidelines [32]; therefore, the administration rate of adjuvant chemotherapy for Stage II cases was significantly lower. Furthermore, it is important to consider that patients who underestimated their stage due to having < 12 harvested lymph nodes may have been disadvantaged because they should have received adjuvant chemotherapy. In addition, preoperative CEA, CA 19-9, and NLR, which are generally considered prognostic factors, were not selected as prognostic factors in this study.

This study had some limitations. First, this study was not designed to analyze the prognostic factors of obstructive CRC, but as a post hoc analysis of a retrospective multicenter study to compare the survival and perioperative outcomes of BTS using SEMS, TADT, and emergency surgery for LOCRC. Thus, there were cases with missing data, and we did not investigate histopathological factors, such as lymphatic invasion, vascular invasion and perineural invasion. Second, patients who died because of SEMS placement and TADT placement remained beyond the scope of the present study. However, as no postoperative deaths after SEMS placement have been reported in any previous report on SEMS as BTS, the influence of this shortcoming is expected to be negligible. Third, long-term outcomes were assessed using RFS as the primary endpoint in this study. Because of the significant advances in therapeutic chemotherapy in recent years, we believe that there would be only a small difference in long-term outcomes based on OS.

## Conclusion

Similar to those in nonobstructive colorectal cancer, depth of invasion and lymph node metastasis were prognostic factors in LOCRC, and patients with <12 dissected lymph nodes experienced stage migration. This stage of migration may result in disadvantages, such as not being able to receive adjuvant chemotherapy.

## Data Availability

The datasets used and/or analyzed during the current study are available from the corresponding author on reasonable request.

## Abbreviations

CRC: Colorectal cancer
BTS: Bridge to surgery
LOCRC: Left-sided obstructive colorectal cancer
SEMS: Self-expandable metallic stent
RFS: Relapse-free survival
CROSS: ColoRectal Obstruction Scoring System
TADT: Transanal decompression tube
CEA: Carcinoembryonic antigen
CA 19-9: Carbohydrate antigen 19-9
NLR: Neutrophil-to-lymphocyte ratio
N0: Lymph node metastasis-negative
OS: Overall survival
DFS: Disease-free survival
